# Immunomodulatory Effects of Histone Deacetylation Inhibitors in Graft-vs.-Host Disease After Allogeneic Stem Cell Transplantation

**DOI:** 10.3389/fimmu.2021.641910

**Published:** 2021-02-24

**Authors:** Xiaoxiao Xu, Xiaoqin Li, Yanmin Zhao, He Huang

**Affiliations:** ^1^Bone Marrow Transplantation Center, The First Affiliated Hospital, School of Medicine, Zhejiang University, Hangzhou, China; ^2^Institute of Hematology, Zhejiang University, Hangzhou, China; ^3^Zhejiang Engineering Laboratory for Stem Cell and Immunotherapy, Hangzhou, China; ^4^School of Medicine, Zhejiang University, Hangzhou, China

**Keywords:** allo-reactive T cells, allogeneic hematopoietic stem cell transplantation, epigenetic regulation, graft-vs.-host disease, histone deacetylase inhibitors

## Abstract

Histone deacetylase inhibitors are currently the most studied drugs because of their beneficial effects on inflammatory response. Emerging data from numerous basic studies and clinical trials have shown that histone deacetylase inhibitors can suppress immune-mediated diseases, such as graft-vs.-host disease (GVHD), while retaining beneficial graft-vs.-leukemia (GVL) effects. These drugs prevent and/or treat GVHD by modifying gene expression and inhibiting the production of proinflammatory cytokines, regulating the function of alloreactive T cells, and upregulating the function and number of regulatory T cells. Some of these drugs may become new immunotherapies for GVHD and other immune diseases.

## Introduction

Allogeneic hematopoietic stem cell transplantation (allo-HSCT) is one of the most effective therapies for hematological malignancies. Although the overall effect of allo-HSCT has improved with the improvement in conditioning regimens, effective control of infection, HLA matching technology, and donor selection, the incidence of graft-vs.-host disease (GVHD) is still 30–60%, with a mortality rate of 30–50% (1). GVHD is the main cause of death after transplantation (2), which limits the success of allo-HSCT. Acute GVHD (aGVHD) has been reported to mainly involve the skin, liver, and gastrointestinal tract of patients within 100 days after transplantation. Chronic GVHD is usually diagnosed after day 100 and mainly manifests as autoimmune symptoms, including dry syndrome, scleroderma and obliterative bronchitis. The pathogenesis of GVHD has been confirmed to involve an alloreactive immune response mediated by the activation of donor T lymphocytes (3).

The combination of a calcineurin inhibitor (CNI; i.e., tacrolimus or cyclosporine) plus methotrexate (MTX) and/or mycophenolate mofetil (MMF) is a standard GVHD prophylaxis regimen used with posttransplantation cyclophosphamide (PTCy) in most haploidentical donor transplant (HIDT) protocols (4–6). Alternative immunosuppressive drug combinations may further help reduce the risk of treatment failure. The incorporation of proteasome inhibitors into GVHD-prevention regimens represents one such strategy, which has generated significant interest (7, 8). However, the incidence of aGVHD is still high and CNIs can also inhibit graft-vs.-leukemia (GVL) effect, thereby increasing relapse rate. In addition, currently, methylprednisolone is the first-line treatment for GVHD. However, the probability of complete remission of patients treated with methylprednisolone is <50% (9), and the long-term use of steroids may lead to steroid dependency and steroid-related adverse events, such as infection. Therefore, there is an urgent need to develop a new, safe, and effective strategy for prophylaxis and treatment of GVHD in the field of allo-HSCT.

Histone deacetylase (HDAC) inhibitors (HDACis) are currently used as anticancer drugs. Their effects in immune-mediated diseases have been studied. For instance, butyrate (pan-HDACi) has been reported to inhibit inflammatory response in a murine model of GVHD (10). This paper reviews advances in research on the application of HDACis for GVHD, and discusses their profound implications in immune cells involved in GVHD.

## Overview of Histone Deacetylation Inhibitors

Histones, as structural proteins, are an important component of chromatin. According to the “histone code hypothesis,” specific residues of histone tails exposed to the chromatin surface can be covalently modified, such as through lysine acetylation, to form “histone codes” and then trigger downstream events (11). Histone acetylation level is a result of the interaction between histone acetyltransferases (HATs) and HDACs. These two groups can acetylate or deacetylate histones (mainly H3 and H4) or some specific lysine residues of certain proteins, thereby altering the chromatin structure and ultimately affecting gene expression.

HDACs can be divided into four categories, of which classical classes I, II, and IV have sequence similarity, and their enzyme activities are dependent on Zn^+^. Class I HDACs (HDAC1, 2, 3, and 8) are mainly located in the nucleus, class II HDACs (HDAC4, 5, 6, 7, 9, and 10) often shuttle between the nucleus and cytoplasm, and class IV HDAC (only HDAC11) is mainly located in the nucleus. Class III HDACs are Sir2-related enzymes (SIRT), which are deacetylases that depend on nicotinamide adenine dinucleotide, and this class has seven members, SIRT1–7, which are located in various organelles based on their functions (12, 13).

**Figure 1:**
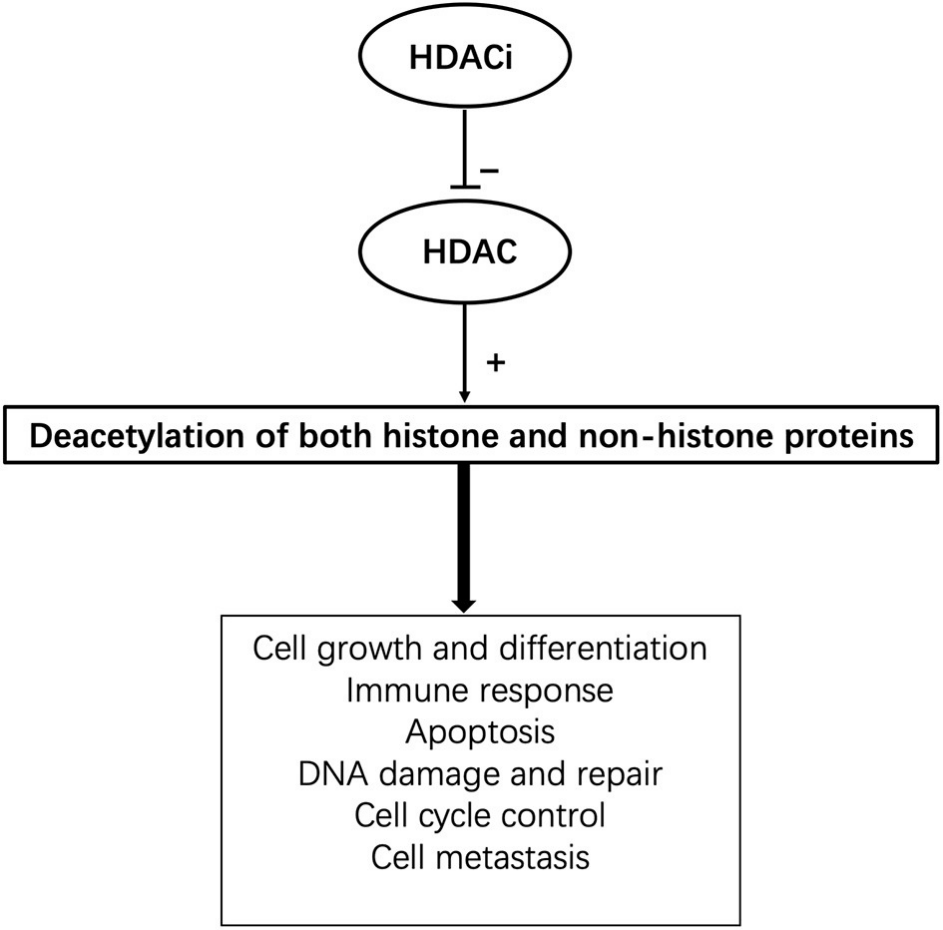
The pleotropic cellular functions of HDACis.

HDACis can inhibit the activity of specific histone deacetylases and upregulate the acetylation level of histones in specific cells as well as other specific non-histone molecules, thus regulating cell growth, differentiation, and immune response. HDACis have different effects on different cells. Their mechanisms include influencing DNA damage, DNA repair, and glycometabolism; altering gene expression; influencing cell growth; and inducing apoptosis, mitosis abnormalities, active oxygen redox, antiangiogenesis, antitumour metastasis, and autophagy of tumors (Figure 1). HDACis can be divided into six categories based on their chemical structure, including hydroxamic acids, short-chain fatty acids, cyclopeptides, electrophilic ketones, benzoamides, and other compounds. At present, four kinds of HDACis have been approved as anti-tumor drugs by the Food and Drug Administration (FDA). Among them, suberoylanilide hydroxamic acid (SAHA) and romidepsin can be used to treat T-cell lymphoma, panobinostat can be used to treat multiple melanomas, and belinostat to treat peripheral T-cell lymphoma (14). Notably, several HDACis have been used in clinical trials for prophylaxis or treatment of GVHD and show dramatic effects, such as a reduction in proinflammatory cytokine secretion and improvement of clinical symptoms (15) (Table 1).

**Table 1:** Summary of the specificities and clinical trials of HDAC inhibitors in GVHD.

**Name**	**Classification**	**HDAC specificity**	**Clinical research stage in GVHD**	**For GVHD**
SAHA	Hydroxamic acids SCFAs	Pan-HDACi	I/II (16, 17)	Prophylaxis
Panobinostat		Pan-HDACi	I/II^1^, ^2^	Treatment
Butyrate	SCFAs	Pan-HDACi	II (18)^3^	Prophylaxis
Romidepsin	Cyclic peptides	Class I and II HDACs (mainly HDAC1 and 2)	I^4^	Prophylaxis
Chidamide	Benzamides	Pan-HDACi	Not reported yet	
Nicotinamide	SIRT inhibitors	Class III HDACs	I/II (19)	Prophylaxis

### Hydroxamic Acids

Hydroxamic acids chelate with metal atoms and bind reversibly with the zinc ions required for HDAC catalytic activity. This type of HDACi, including trichostatin A (TSA), vorinostat (SAHA), panobinostat (PANO), and belinostat, inhibits HDAC activities through competitive binding with the active site. TSA is a natural pan-HDACi that can inhibit class I and II HDACs. It has been used in diverse studies, including *in vitro* and *in vivo* experiments on various cancer strains and immune diseases. TSA reduces the expression of interleukin (IL)-12, interferon γ (IFN-γ), and IL-6 at both the mRNA and protein levels by promoting the acetylation of histones H3 and H4, and ultimately reduces renal disease in lupus mice (20). TSA has also been reported to regulate the expression of various costimulatory/adhesion molecules (such as CD28 and CD154) to alter T-cell function (21). However, the toxicity of TSA limits its clinical application (22). SAHA is a synthetic analog of TSA, but it has significantly lower toxicity. Therefore, SAHA is more widely used than TSA in experimental investigations and clinical applications. SAHA has been shown to prevent GVHD after bone marrow transplantation in mice in an indoleamine-2,3-dioxygenase-dependent manner (23). Another study has also shown that the prevention of GVHD by SAHA is related to the regulation of the inflammatory cytokine environment and the inhibition of signal transducer and activator of transcription 1 (STAT1) (24). The feasibility of SAHA (100 mg, twice a day) combined with tacrolimus and MTX for GVHD prophylaxis after allo-HSCT was evaluated in a prospective phase I/II clinical trial (NCT00810602). Fifty patients diagnosed with high-risk hematological malignant diseases were enrolled in this trial. All the patients had an available 8/8 or 7/8 HLA-matched related donor and underwent reduced-intensity conditioning. The cumulative incidence of grade II–IV acute GVHD by day 100 was 22% (95% confidence interval [CI] 13–36%). The most common non-hematological adverse events included electrolyte disturbances (*n* = 15), hyperglycemia (*n* = 11), infections (*n* = 6), mucositis (*n* = 4), and increased activity of liver enzymes (*n* = 3) (16).

In addition, in a single-center prospective phase II clinical trial, a novel regimen, consisting of SAHA and standard prophylatic drugs, was evaluated after unrelated-donors HSCT. The results showed that the addition of SAHA reduced the incidence of grade II–IV aGVHD on day 100 from 48 to 28% or lower, assuming a type I error of 5%, and enhanced the acetylation of histone H3 in peripheral blood mononuclear cells while reducing IL-6 secretion (median, 4.2 vs. 7.6 pg/mL; *P* = 0.028) (NCT01790568) (17).

PANO has been approved by the FDA as a third-line treatment for multiple myeloma (25). Bug et al.^1^ reported a phase I/II clinical trial of oral maintenance therapy using PANO for patients with myelodysplastic syndrome or acute myeloid leukemia who underwent allo-HSCT. Furthermore, in a phase I/II clinical study (NCT01111526), PANO was used in combination with glucocorticoids for the treatment of GVHD^2^. All participants in this study took PANO at a maximum tolerated dose (5 mg) three times a week for a month. At 36 days after study initiation, complete responses were observed in 12 patients (75%), partial responses were observed in 3 (19%), and progressive disease in 1 (6%). These trials demonstrated that PANO is safe to use after allo-HSCT; moreover, it can control GVHD and additionally function in targeting minimal residual lesions.

### Short-Chain Fatty Acids (SCFAs)

SCFAs cannot bind to Zn^2+^ in the active center of HDAC; therefore, their inhibitory effect on HDAC is weaker than that of hydroxamic acids. SCFAs are the products of bacterial degradation of unabsorbed starch and non-starch polysaccharides (e.g., fibers). They are important anions in the colon's cavity and affect the morphology and function of colon epithelial cells (26). Some studies have shown that SCFAs can be absorbed by the intestinal epithelial cells (IECs), from where they enter the circulatory system through the liver, and ultimately affect cardiovascular function and inflammatory response (27, 28). Acetate, propionate, and butyrate are the main components of SCFAs. Among these, butyrate is the most important HDACi; it inhibits class I and II HDACs and has been shown to inhibit inflammatory responses in various inflammatory models. Cleophas et al. (29) found that butyrate can inhibit the expression of proinflammatory cytokines (IL-1β, IL-6, IL-8, and IFN-γ) in gouty arthritis and has strong anti-inflammatory effects. Furthermore, it has been found that fecal butyrate levels are decreased in patients after allo-HSCT. In a clinical study, stool samples from patients were obtained at baseline (before conditioning regimen), and on day 0 (day of allo-HSCT), day 7 post transplant, and day 14 post transplant. The results showed that the level of butyrate was significantly lower on day 14 than at the baseline, which was collected before allo-HSCT (*P* = 0.0039) (18). In 2016, to verify the effectiveness of butyrate in GVHD prophylaxis, a prospective phase II clinical trial (NCT02763033) was initiated to determine whether resistant starch can reduce the incidence of aGVHD^3^. In their ongoing study, the investigators speculated that the short-term administration of resistant starch increases intestinal butyric acid levels, thereby reducing the incidence of GVHD. In addition, in a previous study by Mathewson et al., (10) butyrate was effective in the treatment of GVHD in a mice model. The reduced butyrate in IECs after allo-HSCT resulted in decreased histone acetylation, whereas butyrate restoration improved the intestinal epithelium junction, re-established the intestinal flora structure, decreased IECs apoptosis, influenced IECs to present major histocompatibility complex (MHC) class II antigens.

### Cyclic Peptides

Cyclic peptides are the most complex class of HDACis. They inhibit the enzyme activity of class I and II HDACs by interacting with Zn^2+^ at HDAC's active sites. Cyclic peptide HDACis can be classified as sulfur-containing and sulfur-free inhibitors. FR235222 is a sulfur-free cyclotetrapeptide inhibitor. It was first isolated from the fermentation broth of Cladosporium (*Acremonium* sp. No. 27082) by Mori et al. (30). They found that FR235222 has a strong immunosuppressive ability, which effectively inhibited the proliferation of T cells and delayed hypersensitivity in mice and adjuvant-induced arthritis in rats. AS1387392 is an analog of FR235222, but it has better pharmacokinetic characteristics and is an orally bioavailable HDACi. Therefore, AS1387392 can be used as a new and effective immunosuppressant (31). Romidepsin is a sulfur-containing peptide HDACi, with a unique ring structure. It has been approved by the FDA for the treatment of cutaneous T-cell lymphoma (32). In addition, recent studies have found that romidepsin can inhibit the activation of STAT1 and STAT3 by inducing suppressor of cytokine signaling 1 expression, and it can suppress the expression of proinflammatory cytokines (e.g., IL-1β) induced by sodium urate crystals (33). In an ongoing phase I clinical trial (NCT02512497), romidepsin was administered in combination with fludarabine and busulfan before and after allo-HSCT to verify whether it helps in controlling leukemia or lymphoma and evaluate the safety of this combination^4^.

### Benzamides

Entinostat (MS-275) is a typical synthetic benzamide HDACi that selectively inhibits class I HDAC enzyme activity. Saito et al. (34) first discovered that MS-275 has a pronouned antitumour activity in mice. In addition to this activity, MS-275 has been used as an effective anti-inflammatory agent in recent studies and has been verified to be effective in some inflammatory models, such as experimental autoimmune encephalomyelitis and rheumatoid arthritis (35, 36). Lin et al. (35) found that MS-275 can effectively improve collagen-induced arthritis in animal models of rheumatoid arthritis; considerably reduce claw swelling, bone erosion, and absorption; and reduce serum IL-6 and IL-1β levels. Moreover, another study found that MS-275 can effectively inhibits inflammatory response in experimental autoimmune neuritis (EAN) in rats by inhibiting inflammatory T cells, macrophages, and proinflammatory cytokines, and inducing anti-inflammatory immune cells and molecules. This indicates that MS-275 may be an effective candidate drug for treating autoimmune neuropathy (36).

Chidamide is an orally absorbed benzamide pan-HDACi that was independently developed in China. It can stimulate the expression of Foxp3, a key transcription factor of regulatory T (Treg) cells, in patients with idiopathic thrombocytopenic purpura (ITP) and in ITP model mice. Moreover, it upregulates the expression of intracellular cytotoxic T-lymphocyte-associated protein 4 in Treg cells, induces Treg cell expansion, and restores immune tolerance (37). However, it is still worth exploring further whether benzamides HDACi can effectively prevent GVHD.

### Sirtuin (SIRT) Inhibitors

Sirtuin inhibitors include nicotinamide, which inhibits all class III HDACs, and specific SIRT1/2 inhibitors, such as sirtinol, cambinol, and EX-527. Nicotinamide can inhibit proliferation and induce the apoptosis of chronic lymphoblastic leukemia cells by activating the p53/miR-34a/SIRT1 network (38). In a phase I/II clinical trial, it was confirmed that nicotinamide can be used to expand umbilical cord blood cells *in vitro*, and the median recovery times of neutrophils and platelets were shortened by 9.5 days (95% CI, 7–12 days) and 12 days (95% CI, 3–16.5 days), respectively, after umbilical cord blood transplantation, which significantly improved the safety of cord blood transplantation and reduced the incidence of GVHD (19). Anusara et al. (39) found that SIRT-1 knockout in mice enhanced p53 acetylation in T cells and promoted Treg stability. Furthermore, selective inhibition of SIRT1 by EX-527 significantly alleviated GVHD, improved survival of the mice, and preserved the GVL effect mediated by donor T cells.

## Effects of HDACis on GVHD

**Figure 2:**
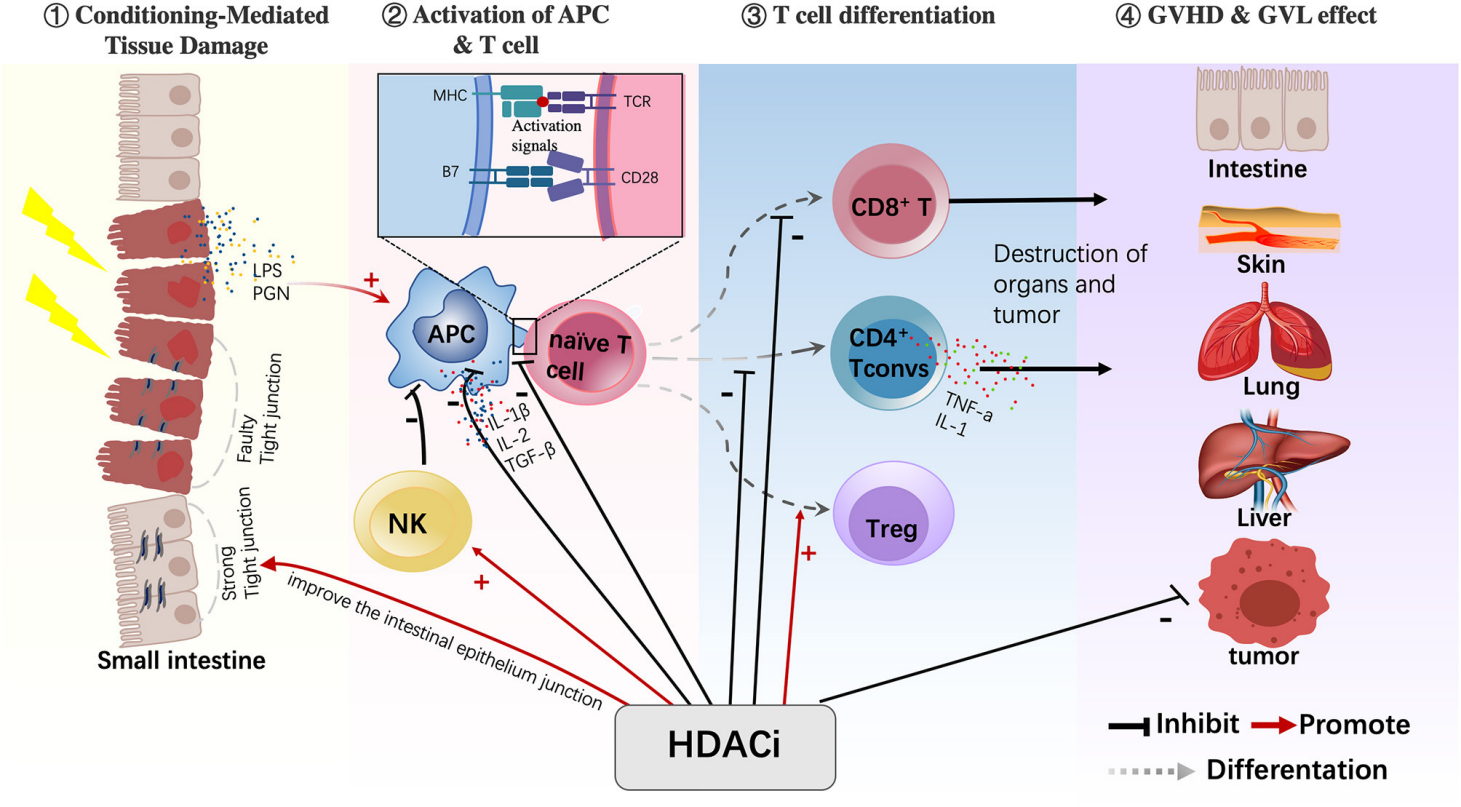
HDACis have various immunomodulatory effects on different cells. HDACis play an important role in regulating the maturation of APCs, reducing their antigen-presenting capacity and inhibiting the production of proinflammatory cytokines. HDACis also promote the conversion of naive T cells into Tregs and increase their function. In addition, HDACis activate NK cells and inhibit CD4^+^ Tconv cells and CD8^+^T cells. Moreover, HDACis can improve the intestinal epithelium junction during the GVHD process (HDACi, histone deacetylase inhibitor; APC, antigen-presenting cell; Treg, regulatory T cell; NK, natural killer cell; CD4^+^ Tconv, CD4^+^ conventional T cells).

The pathological mechanism of GVHD has now mostly been clarified and can be divided into three stages. In the first stage, tissue damage is caused by conditioning chemotherapy or infection, which activates “dangerous signal pathways” and leads to the secretion of proinflammatory cytokines (such as IL-1 and TNF-α). The second stage is the activation and amplification of effector T cells, in which antigen-presenting cells (APCs) and proinflammatory cytokines jointly activate donor T cells and cause their proliferation and the secretion of proinflammatory cytokines. The third stage is the immune effect stage, in which the activated donor T cells and abundant proinflammatory cytokines lead to tissue damage of the host, and this organ injury can further activate T cells. The following section summarizes the possible mechanism of action of HDACis in the treatment of GVHD in terms of occurrence and development stages of GVHD (Figure 2).

### HDACis Inhibit the Production of Inflammatory Mediators

Reducing the secretion of proinflammatory cytokines, thereby downregulating immune response, is an effective strategy for the prophylaxis and treatment of GVHD. ITF2357 has been reported to decrease the expression of various mRNAs induced by IL-1β, including those of cytokines (IL-6 and IL-8), chemokines (CXCL2, CXCL5, CXCL6, and CXCL10), matrix-degrading enzymes (MMP1 and ADAMTS1), and other inflammatory mediators, and promote the degradation of IL-6, IL-8, PTGS2, and CXCL2 mRNAs (40). SAHA, which has been approved by FDA for the treatment of T-cell lymphoma, has antitumor effects at micromolar concentrations, whereas a nanomolar SAHA concentration can reduce the secretion of inflammatory cytokines, such as IFN-γ, TNF-α, IL-1, and IL-12. SAHA can downregulate the mRNA levels of TNF-α and IFN-γ by enhancing the acetylation level of histone H3 and inhibit the secretion of inflammatory cytokines (41, 42). Moreover, butyrate can enhance the acetylation of TNF-α and IL-6 promoters and block the binding of RNA polymerase II with TNF-α and IL-6 gene promoters. In other words, transcription initiation is inhibited, and the expression of TNF-α and IL-6 is reduced (43).

### HDACis Regulate the Function of APCs

Dendritic cells (DCs) are the most effective type of APCs; they play an important role in the pathological process of GVHD. On the one hand, DCs can activate donor T cells by presenting host antigens; on the other hand, they can secrete numerous pro-inflammatory cytokines to further aggravate tissue damage. Pretreatment with TSA reduces the antigen-presenting activity of lipopolysaccharide (LPS)-induced DCs in a dose-dependent manner. TSA plays a role in regulating the maturation of DC cells; thus, pretreatment of DCs with TSA before LPS stimulation reduces the expression of maturation markers to the same level as that of immature DCs. In addition, TSA reduces the production of IL-2 in mature DCs stimulated by LPS (44). Furthermore, TSA can reduce the levels of proinflammatory cytokines (IL-1β, IL-12, and TGF-β) secreted by DCs (45).

### HDACis and Regulatory T Cells

Sakaguchi et al. (46) first discovered that a small group of CD4+ T cells, named Tregs, expresses high levels of CD25 and that the removal of Tregs leads to autoimmune diseases. Tregs play a key role in maintaining peripheral immune tolerance by preventing autoimmunity and chronic inflammation (46). Subsequently, Hori S et al. demonstrated that Foxp3 is specifically expressed in Tregs and is a key regulator of cell development and function (47). In recent years, preclinical studies have shown that adoptive retransfusion of Tregs can inhibit GVHD and prevent or delay allograft rejection (48).

The acetylation of lysine in Foxp3 is necessary to maintain Treg function. Foxp3 acetylation promotes its binding with the IL-2 promoter and subsequently inhibits endogenous IL-2 production. HDAC can inhibit *FOXP3* gene transcription to some extent, whereas HDACis can enhance the homeostasis mediated by Treg proliferation. Therefore, HDACis are considered to be effective for increasing the number and inhibitory function of Tregs (49). Choi et al. (50) analyzed the immune response of patients receiving vorinostat for GVHD prevention after HSCT. Their results showed increases in the number of Tregs, methylation level of the Treg-specific demethylated region, and CD45RA and CD31 expression on the surface of Tregs, as well as enhanced inhibitory function.

### Effect of HDACis on Natural Killer (NK) Cell Function

Donor NK cells can reduce the occurrence of GVHD by eliminating host APCs and secreting IL-10 in the early stage of transplantation, and they can directly eliminate recipient tumor cells. Delayed expansion of NK cells, especially immature NK cells, is associated with an increased aGVHD incidence and severity. Compared with patients without GVHD, patients with GVHD showed a significant decrease in the number of NK cells in peripheral blood (51). Entinostat enhanced NK cell function through epigenetic upregulation of the IFIT1-STING-STAT4 pathway. In that study, the researchers found that entinostat significantly increased the expression of NKG2D, an essential NK cell-activating receptor. Furthermore, the killing function of NK cells was also enhanced. In terms of its mechanism, entinostat increases the accessibility of the chromatin in the promoter region of interferon-induced protein with tetratripeptide repeats 1 (IFIT1), thus upregulating the mRNA and protein expression levels of IFIT1 and enhancing the IFIT1–STING–STAT4 pathways mediated by IFIT1 (52). However, further studies investigating whether HDACi also promotes the killing function of NK cells by regulating acetylation, thereby eliminating recipient APCs and inhibiting GVHD, are warranted.

### Effect of HDACis on Helper T Cells

As mentioned above, donor-derived T cells are the key cell subsets in the development of GVHD, whereas GVL also requires allogeneic T cells. Th1, Th17, and Th2 subpopulations contribute to GVHD, but they mediate GVHD to different degrees of severity (Th1 and Th17 mediating more severe GVHD) and different distributions of GVHD in target tissues (conversion to Th1 or Th17 cells is related to intestinal GVHD, whereas conversion to Th2 cells is related to lung GVHD) (53). Long et al. (54) demonstrated that valproic acid (VPA) can reduce the incidence and lethality of GVHD after allo-HSCT in mice, which is related to the downregulation of Akt phosphorylation and thus, inhibition of Th1 and Th17. In addition, TSA can inhibit inflammation by increasing the number of Th2 cells and enhancing their ability to secrete IL-4 (55).

## Discussion

This paper reviews the classification of HDACi and their direct or indirect effects on immune cells involved in GVHD. HDACi are an important class of anti-tumor drugs that have been used to treat a variety of tumors. Moreover, an increasing number of basic research and clinical trials have shown that HDACi have a strong anti-inflammatory effect and can negatively regulate GVHD while retaining beneficial GVL effects. Therefore, HDACi may become new immunotherapeutic options for prophylaxis and treatment of GVHD or other immune diseases.

## Author Contributions

Under the supervision of HH and YZ, the manuscript was written by XX and XL. All authors contributed to the article and approved the submitted version.

## Conflict of Interest

The authors declare that the research was conducted in the absence of any commercial or financial relationships that could be construed as a potential conflict of interest.

## Footnotes

^1^ClinicalTrials, n.d., ClinicalTrials.gov. NCT01451268.

^2^ClinicalTrials, n.d., ClinicalTrials.gov. NCT01111526.

^3^ClinicalTrials, n.d., ClinicalTrials.gov. NCT02763033.

^4^ClinicalTrials, n.d., ClinicalTrials.gov. NCT02512497.

## Abbreviations

HDAC: histone deacetylase
GVHD: graft-vs.-host disease
Allo-HSCT: allogeneic hematopoietic stem cell transplantation
HDACi: HDAC inhibitors
HATs: histone acetyltransferases
SIRT: Sir2-related enzymes
NAD: niacinamide adenine dinucleotide
SAHA: suberoylanilide hydroxamic acid
PANO: panobinostat
TSA: trichostatin A
IFN-γ: interferon γ
SCFAs: short-chain fatty acids
IECs: intestinal epithelial cells
MHC: major histocompatibility complex
VPA: valproic acid
Treg: regulatory T
ITP: idiopathic thrombocytopenic purpura
APCs: antigen-presenting cells
NK: natural killer
CD4+ Tconvs: CD4+ conventional T cells
IFIT1: tetratripeptide repeats 1.
